# Cancer stem cells in drug resistance: an introduction to the e-book covering the special issue on the “Cancer Stem Cells and Drug Resistance”

**DOI:** 10.20517/cdr.2023.23

**Published:** 2023-04-26

**Authors:** Balázs Sarkadi

**Affiliations:** Institute of Enzymology, Research Centre for Natural Sciences, Budapest 1117, Hungary.

A major problem in current cancer drug treatment is that a subset of cancer cells in many cases survives even highly efficient chemotherapy, and after a while, these remaining cells cause a recurrence of the disease. Moreover, the re-emerging cancer cells often show a multidrug-resistant phenotype, higher proliferation rate, higher invasiveness, and increased metastatic potential, as compared to original cancer. Cancer relapse and multidrug resistance are widely observed also in the most up-to-date, specifically targeted drug- or immunotherapies, causing an unsurmountable clinical inefficiency in cancer eradication.

A widely favored explanation for this relapse phenomenon is the presence of “stem cell-like” cancer cells, surviving therapy within the original tumor mass and then rapidly generating the recurring malignancy. This presumed tumor-initiating cancer stem cell (CSC) population is suggested to be responsible for increased apoptosis and chemoresistance, higher epithelial-mesenchymal transition (EMT) potential, and clinical cancer relapse and metastasis^[1-6]^. An alternative proposal suggests the presence of drug-tolerant persister cells (DTPC) with special genetic features among the cancer cells, and this drug-tolerant persister (DTP) cell population may be the source of cancer relapse^[7,8]^.

The major conceptual difference between the two proposed sources of re-occurring tumor cells after drug treatment is discussed in detail in the key reviews by Clevers^[9]^ and Borst^[10]^. In very short, the CSC concept envisions within the heterogeneous tumor (containing mostly variably differentiated cells) the presence of specific, drug-resistant, stem cell-like clonal cells. In contrast, the concept of the DTP cells suggests that some of the cells within the tumor mass can transiently and reversibly up-regulate mechanisms responsible for drug resistance, and the surviving cells variably regain their tumor-forming capacity^[7,8]^.

Irrespective of the actual mechanisms, the clonal presence or transient formation of these practically immortal cells poses a major challenge for cancer therapy. Thus, the exploration of the basic biology and the clinical features of these CS or DTP cells may bring a new, successful era in cancer chemotherapy, including targeted therapies. Therefore, an elucidation of the key molecular mechanisms regulating CSC/DTP activities could help the development of novel biomarkers and therapeutic targets to predict or combat the most aggressive malignant tumors in patients^[11-14]^.

The chapters in this special issue e-book aim to explore some of the important features, molecular background, phenotype, and clinical role of the CSCs or DTPCs. Among these, Safa^[15]^ reviews the drug and apoptosis resistance in the tumor-initiating CSCs, how they display increased self-renewal potential, anticancer drugs and radiation resistance, and show an increased epithelial to mesenchymal transition (EMT) progression. Also, the potential specific treatments targeting CSCs are explored in this chapter. Hansen *et al.*^[16]^ discuss the presence of leukemia stem cells in AML and focus on the cell surface markers and intracellular transcription factors that can distinguish these leukemia stem cells from normal hemopoietic stem cells. These biomarkers may significantly help to specifically eliminate the stem cell-like leukemic cells and thus improve leukemia treatment.

The chapter by Gupta *et al.*^[17]^ specifically explores the potential role of CSCs in cancer resistance to immunotherapy. They describe surface markers that are differentially expressed on CSCs and potentially help them to escape from immune surveillance and immune cell-dependent killing. Moreover, they suggest that CSC-released cytokines and other metabolites may result in a decreased anticancer immune response in the tumor microenvironment. All these features may help to devise more effective anticancer immunotherapies.

Koltai^[18]^ in his chapter raises an interesting issue and concept related to the role of pH in the development of the cancer multidrug resistance phenotype, and the potential use of already existing or repurposed drugs to modulate this resistance. In addition, a chapter by Kim *et al.*^[19]^ deals with the potential use of herbal extracts to provide effective treatment in lung cancer, by reducing drug resistance and the growth of cancer stem cell-based tumors from an NSCLC model cell line.

It is hoped that this special issue will provide significant value in enhancing the understanding of the role of cells that have a major impact on clinical drug resistance in cancer.

## DECLARATONS

### Authors’ contributions

The author contributed solely to the article.

### Availability of data and materials

Not applicable.

### Financial support and sponsorship

None.

### Conflicts of interest

The author declared that there are no conflicts of interest.

### Ethical approval and consent to participate

Not applicable.

### Consent for publication

Not applicable.

### Copyright

© The Author(s) 2023.
